# Impact of saturable distribution in compartmental PK models: dynamics and practical use

**DOI:** 10.1007/s10928-016-9500-2

**Published:** 2017-01-03

**Authors:** Lambertus A. Peletier, Willem de Winter

**Affiliations:** 1 Mathematical Institute, Leiden University, PB 9512, 2300 RA Leiden, The Netherlands; 2 Janssen Research & Development, Janssen Prevention Center, Archimedesweg 6, 2333 CN Leiden, The Netherlands

**Keywords:** Saturation, Distribution, Pharmacokinetics

## Abstract

We explore the impact of saturable distribution over the central and the peripheral compartment in pharmacokinetic models, whilst assuming that back flow into the central compartiment is linear. Using simulations and analytical methods we demonstrate characteristic tell-tale differences in plasma concentration profiles of saturable versus linear distribution models, which can serve as a guide to their practical applicability. For two extreme cases, relating to (i) the size of the peripheral compartment with respect to the central compartment and (ii) the magnitude of the back flow as related to direct elimination from the central compartment, we derive explicit approximations which make it possible to give quantitative estimates of parameters. In three appendices we give detailed explanations of how these estimates are derived. They demonstrate how singular perturbation methods can be successfully employed to gain insight in the dynamics of multi-compartment pharmacokinetic models. These appendices are also intended to serve as an introductory tutorial to these ideas.

## Introduction

In practical applications, population pharmacokinetic modellers are regularly confronted with data suggesting nonlinear kinetics of the investigational compound. This may include disproportionate increases in $C_{\rm{max}}$ in single ascending dose (SAD) data or disproportionate accumulation in multiple ascending dose (MAD) data. Such nonlinearities may be difficult to account for using the standard linear compartmental pharmacokinetic (PK) model, even when nonlinear elimination is employed. Here we investigate a class of compartmental PK models which can be characterized as saturable distribution models, which we feel can provide an additional tool enabling pharmacometric modelers to tackle observed nonlinearities in their data.

Compartmental PK models usually combine a central or plasma compartment, which represents the site at which pharmacokinetic sampling takes place, with one or more peripheral or tissue compartments. Such multi-compartmental models typically assume that drug enters the blood stream in the central compartment, is distributed from there via linear first order processes to the peripheral compartments, and finally is eliminated again from the central compartment via either a linear first order process or a saturable Michaelis–Menten process (see e.g. Wagner et al. [1] and more recently, Wu et al. [2], Brocks et al. [3] and Scheerens et al. [4]). While linear distribution from central to peripheral may often provide an adequate description of the observed PK, very few processes in biology are truly linear. Most, if not all biological processes are saturable and may only appear linear because their maximum capacity has not been approached in the observed data. It follows that the standard multi-compartmental PK model with linear distribution can be seen as a special case of a more general class of multi-compartmental PK models with saturable distribution.

Snoeck et al. [5] first developed a population PK model with saturable distribution to account for the nonlinear PK of draflazine. This nonlinearity was found to be related to a capacity-limited, high-affinity binding of draflazine to nucleoside transporters located on erythrocytes and endothelial tissue, and could not be accounted for by conventional, linear distribution PK models. In the model developed by Snoeck et al., draflazine was distributed from a central compartment with linear elimination to three peripheral compartments, two of which were capacity-limited with different capacities but similar affinity and were thought to represent the specific binding of draflazine to its receptors on erythrocytes and tissue, respectively. This model was found to satisfactorily predict the nonlinear, dose-dependent PK of draflazine and its disposition in whole blood and plasma.

In an unpublished study, the approach developed by Snoeck et al. was used to model the PK of compound X, which also showed a markedly nonlinear PK and was also known to bind specifically to receptors on the erythrocytes.

**Fig. 1:**
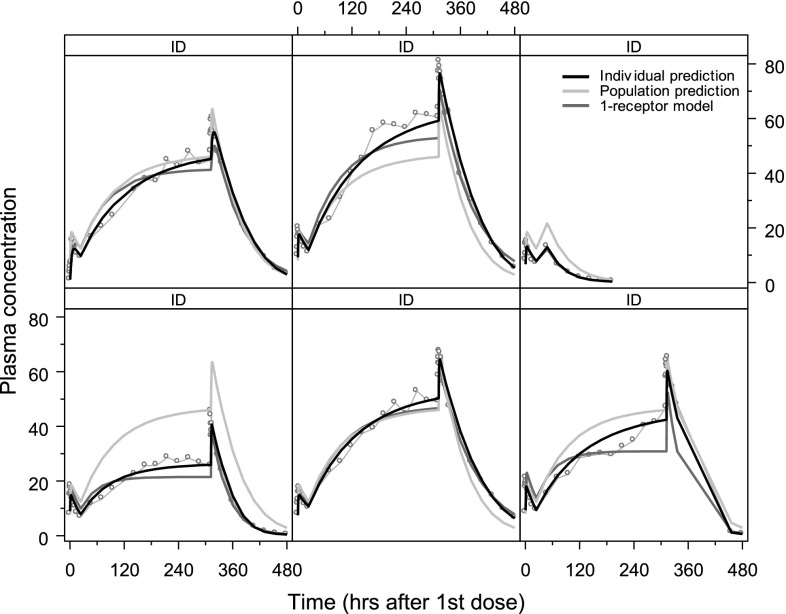
Individual plasma concentration versus time profiles for six subjects receiving a once-daily oral 1500 mg over a period of 3 weeks. The cyan dots show the observed plasma concentrations, the *black curve* shows the individual fit and the *grey curve* the population fit of the 2-receptor model, while the *magenta curves* show the individual fits of the 1-receptor model

Starting from a conventional three compartment PK model, transformation of one of the two peripheral compartments to a low capacity, high affinity compartment with saturable distribution resulted in a highly significant improvement of the model fit. This compartment was thought to represent specific binding to the receptors on the erythrocytes, and addressed a nonlinear dose-dependent increase of $C_{\rm{max}}$ observable in single ascending dose (SAD) studies. However, Fig. 1 shows that this 1-receptor model still failed to address nonlinear dose-dependencies in both accumulation and time to steady-state in multiple ascending dose (MAD) studies. Transformation of the second peripheral compartment to a very high capacity, low affinity compartment with saturable distribution addressed this problem and yielded a further, highly significant improvement of the model fit. This 2-receptor saturable distribution model was used to develop a successful individual dose titration protocol, and was mathematically analysed by Peletier et al. [6].

What kind of non-linearities in the observed PK can be addressed by saturable distribution models, when and how should we apply them? In the following we address such questions by exploring the dynamics of a two-compartmental model with a saturable, Michaelis–Menten type rate function for the distribution of drug from the central to the peripheral compartment. We do this for two opposing variants of saturable distribution: first, we explore the dynamics of a model with a low affinity, high capacity distribution process, and then discuss the dynamics of a model with high affinity, low capacity distribution. In order to assess the impact of saturation, we analyse the dynamics of two classes of models: one with linear and one with saturable distribution.

The objectives of this paper are

(i) To identify characteristic properties of the time courses in the central compartment, and identify differences between linear and saturable models which may serve as *handles* to determine which class of models should be used to fit a given set of data.
(ii) To study the dynamics of the nonlinear model incorporating saturation with a view to understand the impact of the relative capacities and the rate constants of the system and identify the characteristic time-scales.
(iii) To identify the impact of saturable distribution in practical applications, such as the exposure resulting from SAD and MAD regimens.

The mathematical analysis that is used to prove the results in this paper is presented in three appendices. The first two are devoted to the large capacity case, the linear model and the saturable model, and the third appendix is devoted to the small capacity case. The analysis relies strongly on applications of singular perturbation theory (cf. [7, 8]). The appendices are written so that they can be used as a tutorial for applications of this method in pharmacokinetics and pharmacodynamics.

## Methods

In order to study the impact of saturation we compare the dynamics of two distribution models, one with linear and one with nonlinear distribution that involves saturation. In both models a test compound or drug is supplied to an absorption space (1). The drug is then discharged into a central compartment (2), distributed over a peripheral compartment (3), as well as eliminated from the central compartment.

### Linear distribution model

This is the standard linear two-compartment distribution model in which drug flows between the central compartment (1) and the peripheral compartment by diffusion in which the flux is proportional to the difference of the concentrations in the two compartments.

The amount of drug in the absorption space is denoted by $A_1$ and the concentrations in the central and the peripheral compartment are denoted by, respectively, $C_2$ and $C_3$. These quantities satisfy the following system of differential equations:

1 $$ \left\{ \begin{array}{l} \frac{dA_1}{dt}\, = q - k_{a}A_1 \\  V_2\frac{dC_2}{dt}\, = k_{a} A_1 - Cl\times C_2 - Cl_d(C_2 -C_3)\\ V_3\frac{dC_3}{dt}\, = Cl_d(C_2 -C_3)  \end{array} \right. $$

Here *q* denotes the infusion rate, $k_a$ a first order rate constant, *Cl* the non-specific clearance, $Cl_d$ the intercompartmental distribution and $V_2$ and $V_3$ the volumes of the central- and the peripheral compartment.

In comparing this linear distribution model to the nonlinear model involving saturation below, it is convenient to use the amount of drug in the central compartment ($A_2 = V_2 \times C_2$) and in the peripheral compartment ($A_3=V_3 \times C_3$). Introducing these amounts into the system (1) then results in the following system of differential equations:

2 $$ \left\{ \begin{array}{l} \frac{dA_1}{dt} = q - k_{a}A_1 \\ \frac{dA_2}{dt} = k_{a} A_1 - k_{20}A_2 - H \times k_{p}A_2 + k_{p}A_3\\ \frac{dA_3}{dt} = H\times k_{p}A_2 - k_{p}A_3 \end{array} \right.$$

where

$$\begin{aligned} k_{20}=\frac{Cl}{V_2}, \qquad k_{p}=\frac{Cl_d}{V_3} \qquad {\text {and}} \qquad H = \frac{V_3}{V_2} \end{aligned}$$

and *H* is a dimensionless constant which can be viewed as a measure of the “relative capacity” of the central and the peripheral compartment.

### Nonlinear or saturable distribution model

In this model the transfer from the central compartment to the peripheral compartment is saturable, whilst that from the peripheral back to the central compartment is linear. Specifically we study the model

3 $$ \left\{ \begin{array}{l} \frac{dA_1}{dt} = q - k_{a}A_1 \\ \frac{dA_2}{dt} = k_{a} A_1- k_{20} A_2- B_{\rm{max}}k_{p} \frac{A_2}{K_{M}+A_2} + k_{p} A_3 \\ \frac{dA_3}{dt} = B_{\rm{max}}k_{p} \frac{A_2}{K_{M}+A_2} - k_{p} A_3  \end{array} \right. $$

where $q, k_a, k_{20}$ and $k_{p}$ are as in the linear problem. Here $B_{\rm{max}}$ is referred to as the capacity of the peripheral compartment and $K_M$ the Michaelis–Menten constant. Both $B_{\rm{max}}$ and $K_M$ have the dimension of an amount. Thus, saturation is modelled by a Michaelis–Menten term which involves two new parameters, the capacity $B_{\rm{max}}$ and $K_M$. This model has *five* parameters whereas the linear model has *four*.

#### *Remark*

For values of $A_2$ which are small relative to $K_M$, the Michaelis–Menten term in the nonlinear system may be approximated by $(B_{\rm{max}}/K_M)k_p A_2$. Thus the relative capacity *H* in the linear system may be compared to the quotient $B_{\rm{max}}/K_M$ in the nonlinear system.

In the large capacity case, the infusion rate *q* is assumed to be constant, and initially the system is assumed to be empty, i.e., the amounts in the compartments are all assumed to be zero:

4 $$\begin{aligned} A_1(0)=0, \quad A_2(0)=0 \quad {\text {and}} \quad A_3(0)=0 \end{aligned}$$

In the small capacity case, the infusion rate *q* is assumed to be zero, and the initial conditions after an *iv* dose *D* are given by

5 $$\begin{aligned} A_1(0)=D, \quad A_2(0)=0 \quad {\text {and}} \quad A_3(0)=0 \end{aligned}$$

## Steady state

For reference we give here the steady state values of $A_1, A_2$ and $A_3$ when $A_1$ is supplied to the absorption space at a constant rate $k_f(t) \equiv q$. Equating the temporal derivatives in Eqs. (2) and (3) to zero we obtain the following expressions for the steady state amounts $A_{i;{\rm ss}}$ ($i=1,2,3$):

6 $$\begin{aligned}
&A_{1;{\rm{ss}}} = \frac{q}{k_{a}}, \quad A_{2;{\rm{ss}}} = \frac{q}{k_{20}}, \quad A_{3;{\rm{ss}}} = H \times \frac{q}{k_{20}} &&\quad {\text {Linear model}} \\ &A_{1;{\rm{ss}}} = \frac{q}{k_{a}}, \quad A_{2;{\rm{ss}}} = \frac{q}{k_{20}}, \quad A_{3;{\rm{ss}}} = B_{\rm{max}} \frac{q}{q+K_{M} \times k_{20}} &&\quad {\text {Nonlinear model}} \end{aligned}$$

Thus, we can write $A_{3;{\rm ss}}$ in terms of $A_{2;{\rm ss}}$:

7 $$ A_{3;{\rm{ss}}} = H \times A_{2;{\rm{ss}}} \,{\text {({Linear})}}\quad {\text {and}}\quad A_{3;{\rm{ss}}} = B_{\rm{max}} \frac{A_{2;{\rm{ss}}}}{A_{2;{\rm{ss}}}+K_{M}} \,{\text {({Nonlinear})}} $$

We conclude that in *both* models $A_{1;{\rm ss}}$ and $A_{2;{\rm ss}}$ are the same and increase *linearly* with the infusion rate *q*. In the linear model the amount $A_{3;{\rm ss}}$ in the peripheral compartment also increases linearly with *q*, but in the nonlinear model it increases nonlinearly and converges to the capacity $B_{\rm{max}}$ as the infusion rate tends to infinity:

8 $$\begin{aligned}\lim _{q \rightarrow \infty } A_{3,{\rm{ss}}} & = B_{\rm{max}} \end{aligned}$$

We shall see however that whereas in the linear model the time needed for $A_2(t)$ to reach steady state is independent of *q*, in the nonlinear model it varies with the infusion rate.

Evidently, in the absence of an infusion rate, i.e., when $q=0$, the steady state is given by $(A_1,A_2,A_3)=(0,0,0)$.

We contrast the dynamics of models with *large capacity* peripheral compartment, combined with *slow transfer* with models with *small capacity* peripheral compartments endowed with *rapid transfer*.

### Large capacity and slow distribution

We assume,

#### A.1

The capacity of the peripheral compartment is large compared to that of the central compartment.

#### A.2

The drug flows back from the peripheral compartment into the central compartment at a much smaller rate than it is eliminated from the central compartment. Specifically, in terms of the rate constants we assume that:

9 $$\begin{aligned} k_a \gg k_{20} \gg k_p \end{aligned}$$

### Small capacity and rapid distribution

We assume,

#### A.3

The capacity of the peripheral compartment is small compared to that of the central compartment.

#### A.4

Elimination from the central compartment is much slower than the rate with which the drug flows back into the central compartment.

10 $$\begin{aligned} k_a \gg k_{p} \gg k_{20} \end{aligned}$$

## Simulations

In order to acquire a qualitative understanding of the structure of the dynamics of both models, given the relative magnitudes of the rate constants $k_a$, $k_{20}$ and $k_p$, and the capacity of the peripheral compartment of the linear model (*H*) and the nonlinear model ($B_{\rm{max}}$), we perform a series of simulations. We do this separately for the large and the small capacity peripheral compartment.

### Large capacity and slow distribution

We select a series of different values of the infusion rate *q* in order to demonstrate the differences between the linear and the nonlinear model. These simulations will then be done for the following parameter values:

**Table 1:** Parameters values for the linear and the nonlinear model, (2) and (3)

Model	$k_{a}$	$k_{20}$	$k_{p}$	*H*	$B_{\rm{max}}$	$K_{M}$	*q*
Linear	10	0.01	0.0001	100	–	–	1, 2, 3, 4, 5
Nonlinear	10	0.01	0.0001	–	$3 \times 10^4$	100	1, 2, 3, 4, 5
	h$^{-1}$	h$^{-1}$	h$^{-1}$	–	mg	mg	mg h$^{-1}$

Because of the large value of $k_a$, the compound in the absorption space very quickly reaches a quasi-steady state so that we may put $A_1(t)= A_{1,{\rm ss}}= q/k_{a}$ for $t>0$. Thus, the dynamics of the system is effectively determined by the interaction between the central and the peripheral compartment.

**Fig. 2:**
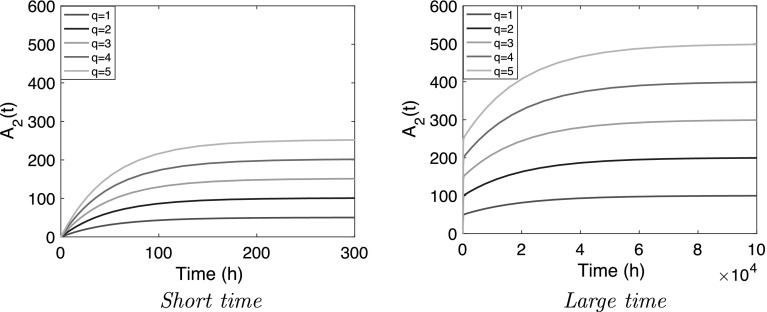
*Linear model* (2) graphs of $A_2(t)$ for increasing infusion rates q $=$ 1, 2, 3, 4, 5 mg h$^{-1}$ when $k_a=10$, $k_{20}=10^{-2}$, $k_p=10^{-4}$ h$^{-1}$ and $H=100$

**Fig. 3:**
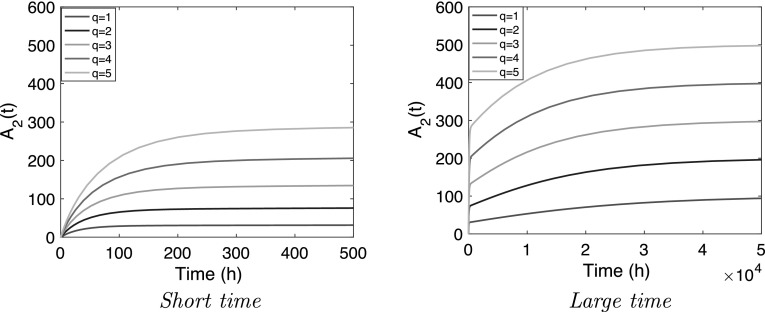
*Nonlinear model* (3) graphs of $A_2$ versus time for the parameter values $k_a=10$, $k_{20}=10^{-2}$, $k_p=10^{-4}$ h$^{-1}$, $B_{\rm{max}}=3 \times 10^4$ mg, $K_M=10^2$ mg

In Figs. 2 and 3 we show how in the linear and the nonlinear model the amount of compound in the central compartment ($A_2$) evolves with time for the different infusion rates. The simulations for the linear and the nonlinear system look similar. Both exhibit a clear two-phase structure, which can be divided into:

$\bullet$ A brief initial phase in which $A_2$ climbs to what appears to be a plateau. We shall refer to this value of the amount of compound as the *Plateau value* and denote it by $\overline{A}_2$.

$\bullet$ A second, much longer phase in which the final plateau value $\overline{A}_2$ of the first phase serves as a starting point of a slow rise towards the final limit which, as expected, is the steady-state value $A_{2;{\rm ss}}$.

However, Figs. 2 and 3 demonstrate that the impact of the infusion rate *q* is very different. Here we focus on how the infusion rate *q* affects the following characteristics of the dynamics:

1. The plateau value $\overline{A}_2$ after the first phase.
2. The half-life of the convergence to the plateau value $\overline{A}_2$ as well as the half-life of the convergence to the final steady state value $A_{2;{\rm ss}}$.

As can be expected from a linear problem, we see in Fig. 2 and Eq. (6) that $\overline{A}_2$ and $A_{2;{\rm ss}}$ depend linearly on *q* and that the half lives in the two phases are independent of the infusion rate. The simulations in Fig. 3 demonstrate that for the nonlinear model the influence of the infusion rate *q* is more complex. However, the terminal state $A_{2;{\rm ss}}$ is the same as for the linear problem (cf. (6)) and hence depends linearly on the infusion rate:

11 $$\begin{aligned} A_{2;{\rm ss}} &= \frac{q}{k_{20}} \end{aligned}$$

Thus, in comparing the two models one needs to focus on the *complete temporal profile* i.e., the concentration versus time profile for all time. We make the following observations:

- The *plateau value*, $\overline{A}_2$, increases with increasing *q*. For the linear model $\overline{A}_2$ is seen to increase linearly with *q* (cf. Fig. 2) whilst for the nonlinear model the dependence on *q* appears to be *super-linear*, i.e., $\overline{A}_2$ appears to grow faster than linearly with *q* (cf. Fig. 3).
- The *half-life* in the two phases. As the infusion rate *q* increases, the half-life in the first phase appears to increase whilst the half-life in the second phase appears to decrease.

### Small capacity and rapid distribution

**Fig. 4:**
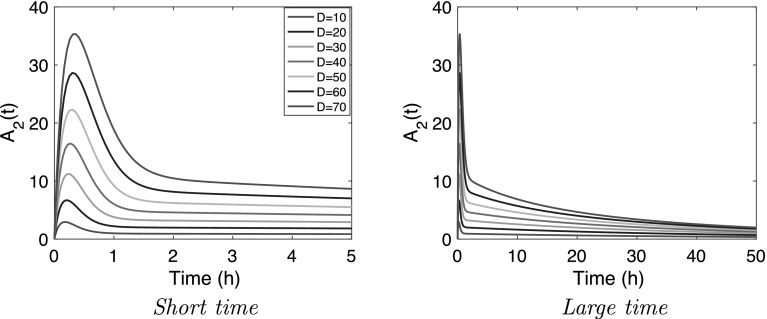
*Nonlinear model* (3) graphs of $A_2$ versus time for $D_0=10,\,20,\ldots ,70$ for the parameter values $k_a=5$, $k_{20}=0.2$ h$^{-1}$, $k_p=1$ h$^{-1}$, $B_{\rm{max}}=100$ mg, $K_M=10$ mg

In Fig. 4 we present a series of simulations for nonlinear, saturable distribution model which exhibit the impact of an *iv* bolus dose on the initial peak of $A_2(t)$. The doses and the parameter values are given in Table 2 in Appendix 2:

**Table 2:** Parameters values for the nonlinear model (3)

Model	$k_{a}$	$k_{20}$	$k_{p}$	$B_{\rm{max}}$	$K_{M}$	$D_0$
Nonlinear	5	0.2	1	100	10	10, 20, ... ,70
	h$^{-1}$	h$^{-1}$	h$^{-1}$	mg	mg	mg h$^{-1}$

It is seen that in this case the disposition also has a two-phase structure: soon after administration, $A_2(t)$ jumps up to a high *Peak value* $A_{2;\rm max}$ quickly drops thereafter (left figure) and then, in a second phase slowly returns to zero (right figure). This Peak value is seen to increase rapidly with *D* in a *super-linear* manner: when *D* increases from 10 to 20 mg then $A_{2; \rm max}$ rises by about 4 mg and when *D* increases from 60 to 70 the rise is about 7 mg, i.e., almost double the low-dose increase.

Thus, as in the large capacity case, the graphs of $A_2$ versus time exhibit a two-phase structure, albeit with a completely different shape. A brief initial phase, say for $0<t < t_0$, in which $A_2(t)$ exhibits a violent up- and down swing which ends with $A_2$ at an intermediate *plateau value* $\overline{A}_2$, followed by a much longer elimination phase.

## Results

Many of the observations made in the simulations can be explained through mathematical analysis of the linear two-compartment model (2) and the nonlinear model (3). Below we present a series of results from such analysis. We discuss the large capacity and the small capacity case in succession.

### Large capacity and slow distribution

At first sight the simulations in Figs. 2 and 3 for the two models are qualitatively similar: a rapid rise of $A_2$ towards an intermediate plateau $\overline{A}_{2}$, the *plateau value*, followed by a slow rise towards the final steady state $A_{2;{\rm ss}}$ given in Eq. (6). In order to discriminate between the dynamics of the linear and the nonlinear model it is therefore important to obtain detailed and quantitative information about characteristics of the dynamics over time. We focus here on two such characteristic properties:

- The intermediate plateau value $\overline{A}_2$, and
- The half-life of the convergence as $A_2$ tends to $\overline{A}_{2}$, and as $A_2$ tends to $A_{2;{\rm ss}}$

and the way these quantities depend on the infusion rate, the capacity and the different rate constants.

For both models we present such quantitative estimates of the plateau value and the half-life in the first and the second phase. Their proofs are given in the mathematical analysis presented in Appendices 1 and 2.

#### Plateau value

The existence of a plateau value is a result of the two-phase structure of the dynamics of this system in which two different time scales can be distinguished:^1^

12 $$\begin{aligned} Short: \,\, t_{1/2}=O(1/k_{20})\quad k_{20}\rightarrow \infty \qquad {\text {and}}\qquad \nonumber \\ Large:\,\, t_{1/2}=O(1/k_{p})\quad k_{p}\rightarrow 0^{4}\, \end{aligned}$$

In light of the basic assumption (9) there is a significant difference between these two time scales. For the parameter values of Table 1 the half-life of the first phase is about a factor 100 shorter than that of the second phase.

During the first phase, return flow from the peripheral compartment is still negligible because $k_p$ is very small and $A_3$ is still building up. Therefore, during this phase the term $k_pA_3$ modelling the back flow from the peripheral compartment into the central compartment may be omitted. Removing this term from the equation for $A_2$ in the systems (2) and (3) yields a *single* differential equation involving $A_2$ only.

– *Linear model:* In the absence of back flow from the peripheral compartment, the amount of compound in the central compartment is governed by the equation

13 $$\begin{aligned} \frac{dA_2}{dt} = q - k_{20}A_2 - H \times k_{p}A_2 \end{aligned}$$

In this equation the input term $k_a A_1$ has been replaced by the infusion rate *q* because, thanks to the large value of $k_a$, within a very short time we have $k_a A_1(t) \approx q$.

The right hand side of Eq. (13) has a unique zero, the *plateau value* $\overline{A}_2$, and it can be shown that

14 $$\begin{aligned} A_2(t) \rightarrow \overline{A}_2 = \frac{q}{k_{20}+ H \times k_{p}} \qquad {\text {as}} \qquad t \rightarrow \infty \end{aligned}$$

Observe that

15 $$\begin{aligned} \overline{A}_2 = \frac{q}{k_{20}+ H \times k_{p}} < \frac{q}{k_{20}} = A_{2;{\rm ss}} \end{aligned}$$

i.e., the plateau value is smaller than the steady state value. Thus, the plateau value can be seen as the starting value of the second phase in which $A_2(t)$ climbs further towards the final value $A_{2;{\rm ss}}$.

##### *Remark*

Because the system (2) is linear, the amounts $A_1, A_2$ and $A_3$ will depend linearly on the infusion rate *q*. This is indeed seen in the expression for the plateau value. Thus,

16 $$\begin{aligned} \frac{1}{q}\times \overline{A}_2 = \frac{1}{k_{20}+ H \times k_{p}} = {\text {Constant}} \end{aligned}$$

– *Nonlinear model:* Without back-flow from the peripheral compartment, the dynamics in the central compartment is now governed by the equation

17 $$\begin{aligned} \frac{dA_2}{dt} = q - k_{20}A_2 - B_{\rm{max}}k_{p} \frac{A_2}{K_{M}+A_2} \end{aligned}$$

and

18 $$\begin{aligned} A_2(t) \rightarrow \overline{A}_2 \qquad {\text {as}} \qquad t \rightarrow \infty \end{aligned}$$

where $\overline{A}_2$ is the unique positive zero of the right hand side of Eq. (17), or of the quadratic equation

19 $$\begin{aligned} A_2^2 - \frac{1}{k_{20}}\left( q- k_{20}\,K_M- k_p \,B_{\rm{max}} \right) \,A_2 - \frac{1}{k_{20}} \,K_M\times q = 0 \end{aligned}$$

Therefore

20 $$\begin{aligned} \overline{A}_2 =\frac{1}{2k_{20}}\left\{ q-k_{20}\,K_M-k_p \,B_{\rm{max}} +\sqrt{\left( q-k_{20}\,K_M-k_p \,B_{\rm{max}}\right) ^2 +4 k_{20}\,K_M \times q}\right\} \end{aligned}$$

**Fig. 5:**
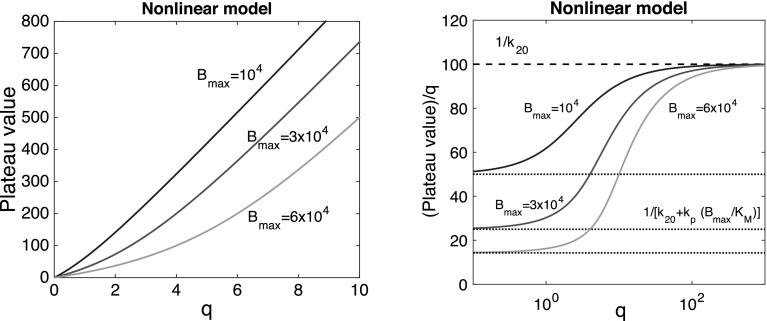
Variation of the plateau value $\overline{A}_2(q)$ (*left*) and the normalised plateau value $\overline{A}_2(q)/q$ (*right*) for the nonlinear model as they vary with *q*, when the data are $k_p=10^{-4}$ h$^{-1}$, $k_{20}=10^{-2}$ h$^{-1}$, the capacity takes the values: $B_{\rm{max}}=10^4$ (*blue*) $3 \times 10^4$ (*red*) and $6 \times 10^4$ (*green*) mg, and $K_M=100$ mg

In Fig. 5 we show how in the nonlinear model, the plateau value $\overline{A}_2$ and the plateau value normalised with respect to the infusion rate $\overline{A}_2/q$ vary with *q*.

In contrast to the linear model, where this quotient is constant, in the nonlinear problem the normalised plateau is seen to be an increasing function of *q*, which connects two asymptotes. Expanding the expression for $\overline{A}_2/q$ in (19) for small and large values of *q*, we find that

21 $$\begin{aligned} \frac{1}{q}\times \overline{A}_2(q) \rightarrow \left\{ \begin{array}{ll} \ell _- \buildrel \rm{def} \over =\frac{1}{k_{20}+ k_p \, (B_{\rm{max}}/K_M)} \qquad &{} \,\, {\text {as}} \,\,q \rightarrow 0 \\ \ell _+ \buildrel \rm{def} \over =\frac{1}{k_{20}} \qquad &{}\,\,{\text {as}}\,\, q \rightarrow \infty \\ \end{array} \right. \end{aligned}$$

The limits $\ell _\pm$ reflect the fact that

1. For large values of $A_2$, i.e., $A_2 \gg K_M$, the saturable nonlinear term is small compared to the linear term $k_{20}\times A_2$ ($B_{\rm{max}} \approx 0$) and the model approximates a linear model with $H=0$.
2. For small values of $A_2$ ($A_2 \ll K_M$), the nonlinear Michaelis–Menten term may be approximated by a linear term: $k_p(B_{\rm{max}}/K_M)\times A_2$ and the model approximates a linear model with $H=(B_{\rm{max}}/K_M)$. The limit obtained in (21) then corresponds with what is seen for the linear model in (14).
3. For any fixed $q>0$, the plateau value $\overline{A}_2$ decreases as the capacity of the peripheral compartment $B_{\rm{max}}$ increases, and^2^
  22 $$\begin{aligned} \overline{A}_2(q, B_{\rm{max}}) \sim \frac{K_M}{k_{20}} \times \frac{q}{B_{\rm{max}}} \qquad {\text {as}} \qquad B_{\rm{max}} \rightarrow \infty \, \end{aligned}$$
4. The small infusion limit in Eq. (21) demonstrates the sensitivity of the plateau value to changes in $B_{\rm{max}}$.

#### Conclusion

 The simulations shown in Fig. 5, together with the analytical estimates derived from the model equations provide valuable diagnostic tools for identifying saturable elimination. Increasing the infusion rate we observe (i) An increasing plateau value which, when normalised by the infusion rate *q*, is still increasing and is uniformly bounded above and below by positive limits $\ell _{\pm }$. (ii) Simple explicit expressions for $\ell _\pm$ which yield quantitative information about $k_{20}$ and $k_p B_{\rm{max}}/K_M$. (iii) Additional estimates for $B_{\rm{max}}$, $K_M$ and $k_p$ can be obtained from the value of *q* at the transition from $\ell _-$ to $\ell _+$.

#### Terminal slope

In both models, the amount of compound $A_2(t)$ in the central compartment converges, in the first phase towards the plateau value $\overline{A}_2$ and then in the second phase towards the steady state $A_{2;{\rm ss}}$. The rate of convergence towards these limits is characterised by the half-life ($t_{1/2}$) or the *terminal slope* $\lambda _z$. We obtain accurate approximations for the terminal slope for each of the models, which we denote by $\lambda _z^{(1)}$ for the first phase and $\lambda _z^{(2)}$ for the second phase, and discuss how $\lambda _z^{(1)}$ and $\lambda _z^{(2)}$ vary with the infusion rate *q* and the capacity *H* or $B_{\rm{max}}.$ –*Linear model* in this model the terminal slope is independent of the infusion rate. We obtain

23 $$\begin{aligned} \left\{ \begin{array}{ll} \lambda _z^{(1)}(H)=k_{20}+H\times k_p \,\,\,&{} for\,\,the\,\,first\,\,phase \\ \lambda _z^{(2)}(H)=\frac{k_p}{1+H {\frac{k_p}{k_{20}}}} \,\,\, &{} for\,\,the\,\,second\,\,phase \\ \end{array} \right. \end{aligned}$$

Thus, as the capacity *H* increases, the terminal slope changes in opposite directions: in the first phase it increases and in the second phase it decreases, i.e.,

24 $$\begin{aligned} \lambda _z^{(1)}(H) \nearrow \quad {\text {and}} \quad \lambda _z^{(2)}(H)\searrow \qquad {\text {as}} \qquad H \nearrow \end{aligned}$$

– *Nonlinear model:* We present the terminal slope in the first phase and in the second phase in succession

For the *first phase* we establish that:

25 $$\begin{aligned} \lambda _z^{(1)} (q, B_{\rm{max}}) = k_{20} + B_{\rm{max}} \, k_p \frac{K_M}{\{K_M+\overline{A}_2(q, B_{\rm{max}})\}^2} \end{aligned}$$

where $\overline{A}_2(q, B_{\rm{max}})$ is the plateau value. We deduce the following properties:

1. As we have seen in Fig. 5, the plateau value $\overline{A}_2$ increases when the infusion rate *q* increases. Hence, it follows from (25) that $\lambda _z(q, B_{\rm{max}})$ is a *decreasing* function of *q*.
2. When $q \rightarrow \infty$, then $\overline{A}_2(q, B_{\rm{max}}) \rightarrow \infty$ and hence, by (25),
  26 $$\begin{aligned} \lambda _z^{(1)}(q, B_{\rm{max}}) \rightarrow k_{20} \qquad {\text {as}} \qquad q \rightarrow \infty \end{aligned}$$
3. When $q \rightarrow 0$, then $\overline{A}_2(q, B_{\rm{max}}) \rightarrow 0$ and hence, by (25),
  27 $$\begin{aligned} \lambda _z^{(1)} (q, B_{\rm{max}}) \rightarrow k_{20} + \frac{B_{\rm{max}}}{K_M} \, k_p \qquad {\text {as}} \qquad q \rightarrow 0 \end{aligned}$$
4. The terminal slope in the first phase $\lambda _z^{(1)}(q, B_{\rm{max}})$ *increases* as $B_{\rm{max}}$ increases. To see this note that according to Fig. 5, the plateau value $\overline{A}_2(q,B_{\rm{max}})$ decreases when the capacity $B_{\rm{max}}$ increases.

For the *Second phase* the terminal slope is well approximated by the formula

28 $$\begin{aligned} \lambda _z^{(2)}(q, B_{\rm{max}}) = k_p \left( 1+B_{\rm{max}}\, k_p\frac{K_M\, k_{20}}{(q+K_M\, k_{20})^2}\right) ^{-1} \end{aligned}$$

The right hand side suggests the following properties:

1. $\lambda _z^{(2)}(q, B_{\rm{max}})$ is an *increasing* function of *q* and a *decreasing* function of $B_{\rm{max}}$.
2. By expanding the expression for $\lambda _z^{(2)}(q, B_{\rm{max}})$ in (28) for small and large values of *q* we obtain
  29 $$\begin{aligned} \lambda _z^{(2)}(q, B_{\rm{max}}) \rightarrow \left\{ \begin{array}{ll} \frac{k_p}{1+ {\frac{B_{\rm{max}}}{K_M}\frac{k_p}{k_{20}}}}\qquad &{}{\text {as}}\qquad q \rightarrow 0 \\ k_p \qquad &{}{\text {as}}\qquad q \rightarrow \infty \\ \end{array} \right. \end{aligned}$$

Note that as $q \rightarrow 0$, the terminal slope $\lambda _z^{(2)}(q, B_{\rm{max}})$ of the nonlinear model approaches that of the linear problem given by (23) with $H=B_{\rm{max}}/K_M$.

**Fig. 6:**
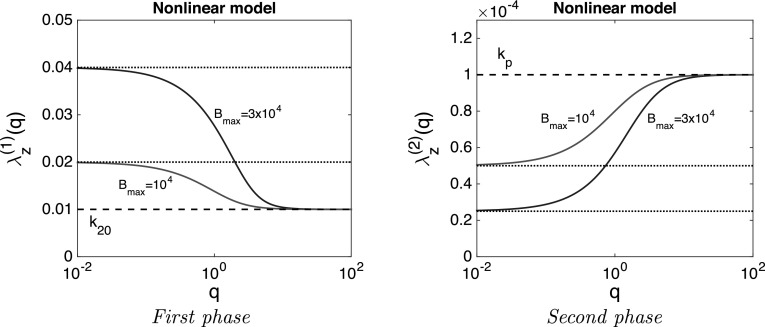
Terminal slopes: $\lambda _{z}^{(1)}(q, B_{\rm{max}})$ (*left*) in the first phase and $\lambda _{z}^{(2)}(q, B_{\rm{max}})$ (*right*) in the second phase versus the infusion rate *q* for the nonlinear model for two values of the capacity: $B_{\rm{max}}=10^4$ (*red*) and $B_{\rm{max}}=3 \times 10^4$ mg (*blue*) and the rate constants $k_a=10$, $k_{20}=10^{-2}$, $k_p=10^{-4}$ h$^{-1}$, and $K_M=10^2$ mg

Figure 6 illustrates and confirms the analytical properties presented above. For the linear model they will be proved in Appendix 1 and for the nonlinear model in Appendix 2.

#### Conclusion

The simulations displayed in Fig. 6, together with analytical expressions for the dependence on *q* of the terminal slope in the first and the second phase are a rich source of information for estimating the different parameters in the models. For both phases, the terminal slope depends monotonically—first down and then up—on *q* and tends to finite non-zero limits as $q \rightarrow 0$ and $q \rightarrow \infty$ which can be computed explicitly.

### Impact of slow leakage from the peripheral compartment

In many practical situations, data are only available for the first phase, and only predictions can be made about the second phase [6]. Clearly, during the long second phase, with its slow dynamics, the influence of leakage from the peripheral compartment may well be relevant. In light of the large capacity of the peripheral compartment this may result in significant losses.

In order to assess the impact of leakage, we modify the nonlinear model and *increase* the first order loss term in the equation for the peripheral compartment by a factor $(1+\alpha )$, where $\alpha > 1$. The equation for $A_3$ in the nonlinear system (3) then becomes

30 $$\begin{aligned} \frac{dA_3}{dt} = B_{\rm{max}}k_{p} \frac{A_2}{K_{M}+A_2} - (1+\alpha )\,k_{p}\, A_3 \end{aligned}$$

whilst the equation for $A_2$, which does not involve $\alpha$, remains the same.

Because it is assumed that $k_p \ll k_{20}$, the two-phase structure is not affected by moderate leakage. And because during the first phase the elimination term in the equation for $A_3$ is small and may be neglected, the first phase will hardly change when some leakage takes place from the peripheral compartment.

On the other hand, during the second phase the impact of leakage will be felt. For instance, leakage has an impact on the steady state values of $A_2$ and $A_3$. They now become:

31 $$\begin{aligned} & A_2 = A_{2;{\rm{ss}}}(\alpha ) \quad {\text {and}} \quad A_{3;{\rm{ss}}} = \frac{1}{1+\alpha }B_{\rm{max}} \frac{A_{2;{\rm{ss}}}(\alpha )}{A_{2;{\rm{ss}}}(\alpha )+K_M} \end{aligned}$$

where $A_{2;{\rm ss}}(\alpha )$ is the root of the quadratic equation

32 $$\begin{aligned} A_2^2 - \frac{1}{k_{20}}\left( q- k_{20}\,K_M- k_p \times \frac{\alpha }{1+\alpha } \,B_{\rm{max}} \right) \,A_2 - \frac{1}{k_{20}} \,K_M\times q = 0 \end{aligned}$$

Note that this equation is the same as Eq. (19) for the plateau value $\overline{A}_2$, except for the factor $\alpha /(1+\alpha )$ which multiplies $B_{\rm{max}}$. An elementary computation shows that

33 $$\begin{aligned} A_{2;{\rm ss}}(\alpha ) \approx \left\{ \begin{array}{ll} \frac{q}{k_{20}} \quad {\text {if}} \, \alpha \ll 1 \\ \overline{A}_2 \quad {\text {if}} \, \alpha \gg 1 \\ \end{array} \right. \end{aligned}$$

Thus, when there is little leakage $(\alpha \ll 1)$, then $A_{2;{\rm ss}}(\alpha )$ is close to the steady state value $A_{2;{\rm ss}}$ given by (6) and when leakage is substantial $(\alpha \gg 1)$, the steady state value drops down to the plateau value $\overline{A}_2$ given by (20).

**Fig. 7:**
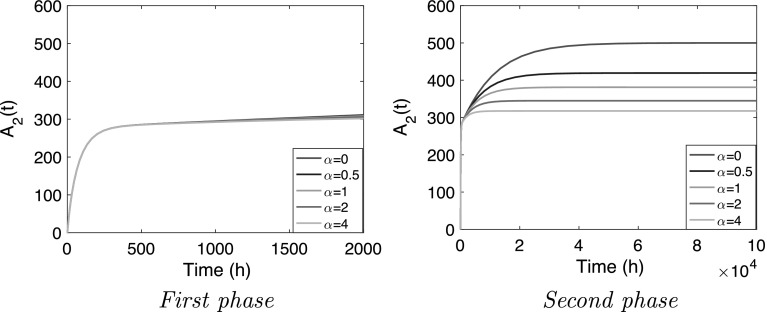
*Nonlinear model with leakage from the peripheral compartment* (3) & (30). Graphs of $A_2$ versus time for $q=5$ and $\alpha =0,\,0.5,\,1,\,2,\,4$ for the parameter values $k_a=10$ h$^{-1}$, $k_{20}=0.01$ h$^{-1}$, $k_p=10^{-4}$ h$^{-1}$, $B_{\rm{max}}=3 \times 10^4$ mg, $K_M=10^2$ mg

In Fig. 7 we show how the temporal behaviour of $A_2$ changes as the elimination from the peripheral compartment increases beyond the original back-flow into the central compartment. The rate of infusion is kept constant ($q=5$) and the elimination is increased from the original value ($\alpha =0$) in four steps to $\alpha = 0.5,\, 1,\, 2$ and 4.

The simulations confirm the analysis: the two-phase structure remains intact, and in the first phase (Fig. 7 left panel) the the additional elimination does *not* show up in the graphs. In the second phase (right panel) elimination does have an impact, and shows a drop in final steady state, starting from the original value ($\alpha =0$) and approaching a value close to the plateau value when $\alpha =4$.

Evidently, the half-life in the second phase decreases as elimination from the peripheral compartment increases.

#### Conclusion

 Elimination is a long-term phenomenon, as is to be expected since it takes place from the peripheral compartment which fills up slowly since $k_p$ is small. Nonetheless, the impact on the central compartment can be significant and, even for moderate elimination rates, can obliterate most of the growth beyond the first phase.

### Small capacity and rapid distribution

To fully appreciate the effect of a large capacity of the peripheral compartment combined with a slow exchange between the two compartments, we conclude with a brief discussion of the dynamics of the nonlinear model for the converse situation: small capacity of the peripheral compartment combined with a fast exchange between the two compartments. Thus, we here assume that

34 $$\begin{aligned} k_a \gg k_p \gg k_{20} \end{aligned}$$

Since in this case the peripheral compartment has small capacity and direct elimination is relatively small, one expects that an *iv* bolus administration will lead to a large peak in concentration in the central compartment. In practical situations the height of this peak can be critical. Thus, to gain insight into this feature we focus here on dynamics after an *iv* bolus dose.

As expected, soon after administration, $A_2(t)$ jumps up to a high *peak value* $A_{2;\rm max}$. This peak value is seen to increase rapidly with $D_0$ in a *super-linear* manner: when $D_0$ increases from 10 to 20 mg then $A_{2; \rm max}$ rises by about 4 mg and when $D_0$ increases from 60 to 70 the rise is about 7 mg, i.e., almost double the low-dose increase.

Thus, as in the large capacity case, the graphs of $A_2$ versus time exhibit a two-phase structure, albeit with a completely different shape. A brief initial phase, say for $0<t < t_0$, in which $A_2(t)$ exhibits a violent up- and down swing which ends with $A_2$ at an intermediate *plateau value* $\overline{A}_2$, followed by a much longer elimination phase.

In order to analyse the dynamics of this system for the parameer values constrained by the conditions (34) and obtain an estimate for $A_{2;\rm max}$ it is necessary to transform the system to dimensionless variables. This analysis, carried out in Appendix 3, yields the following estimates for $A_{2; \rm max}$.

35 $$A_{{2;{\rm{max}}}} (D)\sim \left\{ {\begin{array}{ll}    {M^{{ - M/(M - 1)}}  \times D}\, {{\text{as}}\,D \to 0} \\    {D - M \times K_{M} \ln \left( {\frac{D}{{M \times K_{M}}}} \right)}\, {{\text{as}}\,D \to \infty }\\   \end{array} } \right.\quad M = \frac{{B_{\rm{max}}}}{{K_{M}}} \times \frac{{k_{p} }}{{k_{a}}}$$

Because the initial phase is short and the elimination rate $k_{20}$ is small, the total amount of drug in both compartments is conserved during this initial phase. i.e.,

36 $$\begin{aligned} A_2+A_3 = D \quad {\text {for}} \quad 0 \le t \le t_0 \end{aligned}$$

Because of the larger value of $k_p$ the two compartments are quickly in quasi-steady state, so that after a brief initial adjustment, we may put

37 $$\begin{aligned} & A_3 = \varphi (A_2) \buildrel {\rm{def}} \over = B_{\rm{max}}\frac{A_2}{K_{M}+A_2} \quad {\text {for}} \quad t \ge t_0 \end{aligned}$$

Because Eqs. (36) and (37) both hold at $t_0$, we may use Eq. (36) to eliminate $A_3$ from Eq. (37) to obtain

38 $$\begin{aligned} \overline{A}_2 + B_{\rm{max}}\frac{\overline{A}_2}{K_M + \overline{A}_2} =D \end{aligned}$$

from which we can compute the value of $\overline{A}_2$, right after the initial peak. For small and large dose *D* we find (cf. Appendix 3),

39 $$\begin{aligned} \overline{A}_2(D) \sim \left\{ \begin{array}{ll} \frac{D}{1+(B_{\rm{max}}/K_M)} \,\, {\text {as}} \quad D \rightarrow 0 \\ D-B_{\rm{max}} \quad {\text {as}} \quad D \rightarrow \infty \\ \end{array} \right. \end{aligned}$$

which clearly demonstrates the super-linear behaviour of $\overline{A}_2(D)$. For the terminal slope of the first phase $\lambda _z^{(1)}(B_{\rm{max}})$ we find

40 $$\begin{aligned} \lambda _z^{(1)}(B_{\rm{max}}) =\left\{ \begin{array}{ll} k_a \quad {\text {if}} \quad \frac{B_{\rm{max}}}{K_M}\, k_p > k_a \\ \frac{B_{\rm{max}}}{K_M}\, k_p \quad {\text {if}} \quad \frac{B_{\rm{max}}}{K_M}\, k_p < k_a \\ \end{array} \right. \end{aligned}$$

In order to determine the long time behaviour of $A_2(t)$, we add the equations for $A_2$ and $A_3$ from the system (3). This yields the equation

41 $$\begin{aligned} \frac{d}{dt}(A_2+A_3) = - k_{20}\,A_2 \end{aligned}$$

because $q=0$. We now use the expression for $A_3$ given by Eq. (37), which is valid in the second phase to eliminate $A_3$ from Eq. (41) to obtain

$$\begin{aligned} \frac{d}{dt}\left\{ A_2 + \varphi (A_2)\right\} = - k_{20}\,A_2 \quad {\text {for}}\quad t >t_0 \end{aligned}$$

Using the expression for $\varphi (A_2)$ this equation can be written as

42 $$\begin{aligned} \frac{dA_2}{dt} = - \frac{ k_{20}\,A_2}{1+\varphi '(A_2)} \quad {\text {where}}\quad \varphi '(A_{2;{\rm ss}}) = B_{\rm{max}} \frac{K_M}{(K_{M}+A_2)^2} \end{aligned}$$

where $\varphi '(A_2)$ denotes the derivative of the function $\varphi (A_2)$. The terminal slope $\lambda _z^{(2)}$ of the graph of $A_2$ as it approaches its steady-state value $A_{2;{\rm ss}}$ is given by

43 $$\begin{aligned} \lambda _z^{(2)}(D,B_{\rm{max}}) = \frac{k_{20}}{1+\varphi '(A_{2;{\rm ss}})} \end{aligned}$$

Since $\varphi '(A_2)$ is a decreasing function of $A_2$ it follows that the terminal slope increases as $A_{2;{\rm ss}}$ increases, i.e, as *D* increases. In particular, since $\varphi '(A_2)\rightarrow B_{\rm{max}}/K_M$ as $A_2 \rightarrow 0$, it follows that

44 $$\begin{aligned} \lambda _z^{(2)}(D,B_{\rm{max}}) \rightarrow \frac{k_{20}}{1+(B_{\rm{max}}/K_M)} \qquad {\text {as}}\qquad D \rightarrow 0 \end{aligned}$$

so that $t_{1/2} \rightarrow \{1+(B_{\rm{max}}/K_M)\}\ln (2)/k_{20} \approx 38$ h for the parameter values used in Fig. 4. We see that this estimate is confirmed in Fig. 4.

#### Conclusion

We find that for small capacity and rapid exchange between central and peripheral compartment the dynamics has a brief initial phase followed by a long terminal phase, with an appropriately defined plateau value in between. As in the previous case the terminal slopes yield sensitive markers that can be used to identify the impact of saturation on drug distribution. The plateau value informs about the capacity $B_{\rm{max}}$ and $K_M$, whilst the terminals slope yields estimates for $B_{\rm{max}}$, $K_M$, and about $k_a$ when $(B_{\rm{max}}/K_M)\,k_p>k_a$ and about $k_p$ when $(B_{\rm{max}}/K_M)\,k_p<k_a$.

## Discussion

We have compared the dynamics of two types of models for the distribution of a compound over a central and a peripheral compartment. In one type the elimination of compound from the central compartment into the peripheral compartment is linear, and the other it is saturable and hence nonlinear. In both models, the return flow from the peripheral compartment to the central compartment is linear.

We have focussed on two contrasting extreme cases: (i) In one case, the capacity of the peripheral compartment is large and the back-flow is slow, and (ii) In the other case capacity and back-flow are respectively, small and fast. These cases can be viewed as bench marks in parameter space since they exhibit very different dynamics, each being endowed with its own characteristic ligand versus time graphs.

Both types of graphs exhibit a two-phase structure. However, within these two phases each case has its own characteristic behaviour: the large capacity peripheral compartment retaining ligand for a long time, whilst in the small capacity compartment the presence of ligand, though large, is short-lived.

It is demonstrated that saturable distribution can lead to disproportionately higher steady-state exposures. Specifically:

- In the large capacity/slow distribution case, multiple ascending doses (MAD) yield disproportionately higher steady state exposures.
- In the small capacity/fast distribution case, SAD yield disproportionately higher $C_{\rm{max}}$.

Thus saturable distribution models merit a careful analysis in light of the impact saturation may have on exposure.

In analysing these models subject to the conditions (46–49) listed in Methods, a mathematical framework has been created which can be used to analyse comparable models, which involve additional processes such as (i) leakage, or (ii) binding of the ligand to proteins, lipids and receptors in the central or the peripheral compartment, such as discussed in [9], or (iii) when the model involves additional compartments. This analytical machinery makes it possible to give quantitative estimates of the impact of these processes on the drug distribution between compartments and over time.

As an application of the methods developed in this paper, we show that leakage from the peripheral compartment may have considerable impact over a period of time. If this period extends beyond the period over which measurements are available the need for accurate quantitive predictions is evident.

Distribution over two compartments in which the peripheral compartment has a limited capacity, has much in common with tissue-binding. Here it is the tissue, viewed as a separate compartment, which can become saturated when maximal occupancy is reached. Thanks to this similarity in structure many of the results established in this paper can easily be transposed to the dynamics of tissue-binding.

The mathematical analysis is presented in a series of appendices. They offer an introduction to the use of such methods as (i) the use of dimensionless variables and parameters and (ii) multi-scale analysis. Dimensionless parameters are often a numerical measure of the relative importance of different processes involved in the model, such as direct elimination from the central compartment and distributional transfer between the compartments. Different time-scales are a common occurrence in pharmacokinetics and pharmacodynamics, often due to large differences in concentrations, in rate constants or in binding constants. They make it possible to simplify the often complex systems by means of *singular perturbation theory* (cf. [7, 8]). The appendices demonstrate the practical usefulness of this theory for the study of complex pharmacokinetic and pharmacodynamic systems, and can serve as an introductory tutorial.

In summary, we have demonstrated a number of interesting dynamic properties of saturable distribution models which can be of value in practical modelling applications. In particular, we have shown that such models can account for disproportional accumulation evident in MAD data as well as disproportional increase in $C_{\rm{max}}$ in SAD data. This is achieved by relaxing the assumption of linear distribution in the standard model at the cost of only one extra parameter per peripheral compartment. Saturable distribution models share many properties with models for tissue and receptor binding, which provides another attractive mechanistic underpinning for this class of models. For these reasons, we feel that the saturable distribution model deserves a more prominent place in the pharmacometrician’s toolbox than it currently has. Here we try to promote this by providing a guide to its dynamics and, hence, applicability.

## Appendix 1: mathematical analysis of the linear problem

In Appendices 1 and 2 we focus on the *large capacity - slow distribution case* (cf. 46 and 47). Thus,

45 $$\begin{aligned} k_a \gg k_{20} \gg k_p \end{aligned}$$

and initially $A_i(0)=0$ for $i=1,\,2,\,3$.

In order to compare the “weight” of different terms in the system (2) we introduce dimensionless variables. Thus, we use the steady state values as reference value and put

46 $$\begin{aligned} x=\frac{A_1}{A_{1,{\rm ss}}}, \quad y=\frac{A_2}{A_{2,{\rm ss}}}, \quad z=\frac{A_3}{A_{3,{\rm ss}}} \end{aligned}$$

where $A_{1,{\rm ss}}$, $A_{2,{\rm ss}}$ and $A_{3,{\rm ss}}$ are given by (6). Introducing these dimensionless variables we obtain the system

47 $$\left\{ \begin{array}{l} \frac{dx}{dt} = k_{a}(1-x) \\ \frac{dy}{dt} = k_{20}(x-y)- H \times k_{p}(y - z)\\ \frac{dz}{dt} = k_{p}(y - z)\\ \end{array} \right. $$

This is a linear system, so that on any bounded time interval the solution is bounded.

It remains to introduce a dimensionless time variable. For this purpose we choose three different time scales, each corresponding to one of the rate constants in the system (47).

### A very short time scale

We choose $k_a^{-1}$ h as a reference time and define the dimensionless time $\tau _0 = k_a \, t$. Introducing this time variable into (47) yields the system

48 $$ \left\{ \begin{array}{l} \frac{dx}{d\tau _0} = 1 - x \\ \frac{dy}{d\tau _0} = \nu \,(x-y)- H \times \nu \varepsilon (y - z),\quad \nu = \frac{k_{20}}{k_a}  \quad \varepsilon = \frac{k_{p}}{k_{20}}\\ \frac{dz}{d\tau _0} = \nu \varepsilon (y - z)\\ \end{array} \right.  $$

By assumption (9) the parameters $\nu$ and $\varepsilon$ are small. For the parameter values of Table 1 for the linear problem which are used in Fig. 2, they are: $\varepsilon =10^{-3}$ and $\nu = 10^{-2}$.

It follows that given any finite time interval $0 \le \tau _0<T_0$, then as $\nu \rightarrow 0$, the solution of the system (48) converges uniformly to that of the *Reduced system*:

49 $$ \left\{ \begin{array}{l} \frac{dx}{d\tau _0} = 1 - x \\ \frac{dy}{d\tau _0}= 0\\ \frac{dz}{d\tau _0} = 0\\ \end{array} \right. $$

Remembering that initially, $(x,y,z)=(0,0,0)$, it follows that for $0 \le \tau _0 \le T_0$, the solution of the original system (48) is well approximated by

50 $$\begin{aligned} x(\tau _0)=1-e^{-\tau _0}, \qquad y(\tau _0)=0, \quad {\text {and}} \quad z(\tau _0)=0 \end{aligned}$$

Evidently, $x(\tau _0) \rightarrow 1$ as $\tau _0\rightarrow \infty$. The half-life $\tau _{0;1/2}$ - i.e., the time it takes for $x(\tau _0)$ to reach half of its final value, is equal to $\ln (2)$. Thus, in the original time variable *t*, the half-life is given by $t_{1/2}=\ln (2)/k_a=0.07$ h, which amounts to about 4 min.

### A short time scale

We choose $1/k_{20}$ h as a reference time and define the dimensionless time $\tau _1 = k_{20} \, t$. Introducing this new time variable into the system (47) and putting $x=1$, we obtain the reduced system

51 $$ \left\{ \begin{array}{l} \frac{dy}{d\tau _1} = (1 - y)- \varepsilon \times H (y - z)\\ \frac{dz}{d\tau _1} = \varepsilon (y - z) \end{array} \right. $$

Using phase-plane arguments (cf. [11]) it can be shown that $0<y(\tau _1)<1$ for all $\tau _1>0$. Therefore, $0<z(\tau _1)<\varepsilon$ for all $\tau _1>0$ and hence, since $\varepsilon \ll 1$, we may put $z(\tau _1)=0$, and approximate the first equation of (50) by

52 $$\begin{aligned} \frac{dy}{d\tau _1} = 1-(1+ \varepsilon \times H)\, y \end{aligned}$$

where we have retained the product $\varepsilon \times H$, because it may not be small. The steady state value in this first phase is $y=(1+\varepsilon \times H)^{-1}$; it is the plateau value $\overline{y}$. It follows that

53 $$\begin{aligned} y(\tau _1) \rightarrow \overline{y}= \frac{1}{1+ \varepsilon \times H}\quad {\text {as}} \quad \tau _1 \rightarrow \infty \end{aligned}$$

and the half-life $\tau _{1;1/2}$ in this initial phase are given by

54 $$\begin{aligned} \tau _{1,1/2} = \frac{\ln (2)}{1+ \varepsilon \times H}. \end{aligned}$$

In terms of the original variables we thus obtain

55 $$ \begin{aligned} A_2(t) \rightarrow \overline{A}_2 = \frac{q}{k_{20}}\, \overline{y}= \frac{q}{k_{20}+H\times k_p} \qquad \& \qquad t_{1/2} = \frac{\tau _{1,1/2}}{k_{20}} = \frac{\ln (2)}{k_{20}(1+ \varepsilon \times H)} \end{aligned}$$

When $H=100$ and $k_{20}=0.01$, then $\overline{A}_2=50 \times q$ mg and $t_{1/2} = 34.7$ h (cf. Fig. 2).

### A large time scale

We choose $k_p^{-1}$ h as a reference time and define the dimensionless time $\tau _2 = k_{p} \, t$. Putting this time variable into the system (48) and setting $x=1$ then yields the system

56 $$ \left\{ \begin{array}{l} \varepsilon \frac{dy}{d\tau _2} = 1 - y- \varepsilon \times H \cdot (y - z)\\ \frac{dz}{d\tau _2} = y - z \end{array} \right. $$

Again, by standard singular perturbation theory (cf. [7, 8, 10]) it follows that after a very short time

57 $$\begin{aligned} 1-y- \varepsilon \times H\cdot (y - z) = 0 \qquad \Longrightarrow \qquad y = \frac{1+\varepsilon \times H \times z}{1+\varepsilon \times H} \end{aligned}$$

Using this expression for *y* in the second equation of the system (54), we obtain

58 $$\begin{aligned} \frac{dz}{d\tau _2} = \frac{1 - z}{1+\varepsilon \times H} \end{aligned}$$

Remembering that $z(0)=0$, it follows that

$$\begin{aligned} z(\tau _2)=1-e^{-\gamma \, \tau _2}, \qquad {\text {where}} \qquad \gamma = \frac{1}{1+\varepsilon H} \end{aligned}$$

and the half-life $\tau _{2;1/2}$ in the second phase is given by

59 $$\begin{aligned} \tau _{2,1/2} =(1+\varepsilon \times H)\ln (2) \qquad \Longrightarrow \qquad t_{1/2} = \frac{1}{k_p}(1+\varepsilon \times H) \ln (2) \end{aligned}$$

and the terminal slope $\lambda _z$ is given by

60 $$\begin{aligned} \lambda _z = \frac{k_p}{1+\varepsilon \times H} \end{aligned}$$

Comparing the expressions (55) and (59) for the half-life in, respectively, the first and the second phase, we see that as the capacity *increases*, the half-life *decreases* in the first phase and *increases* in the second phase.

If $H=100$, then $t_{1/2}= 1.4\times 10^4$ h,which agrees with the simulations in Fig. 2.

## Appendix 2: mathematical analysis of the nonlinear model

Here we define the dimensionless variables

61 $$\begin{aligned} x=\frac{A_1}{A_{1,{\rm ss}}}, \qquad y=\frac{A_2}{A_{2,{\rm ss}}}, \qquad z=\frac{A_3}{B_{\rm{max}}} \end{aligned}$$

in which for $A_1$ and $A_2$ we have chosen the same reference values, whilst for $A_3$ we have chosen the fixed capacity $B_{\rm{max}}$ which serves as a uniform bound for $A_3$ (cf. (7)).

In addition we define the dimensionless constants

62 $$\begin{aligned} \beta = \frac{B_{\rm{max}}k_p}{q} \qquad {\text {and}} \qquad \kappa _M = \frac{k_{20}}{q}K_M \end{aligned}$$

Substitution of these variables into the system (3) yields

63 $$ \left\{ \begin{array}{l} \frac{dx}{dt} = k_{a}(1-x) \\ \frac{dy}{dt} = k_{20} (x- y)- \beta k_{20} \left( \frac{y}{\kappa _{M}+y} - z\right) \\ \frac{dz}{dt}= k_{p} \left( \frac{y}{\kappa _{M}+y} - z\right) \end{array} \right.$$

As with the linear model, within a very short time $x(t) \approx 1$ so that we may put $x(t) = 1$ and reduce the system (63) to

64 $$ \left\{ \begin{array}{l} \frac{dy}{dt} = k_{20} (1- y)- \beta k_{20} \left( \frac{y}{\kappa _{M}+y} - z\right) \\ \frac{dz}{dt} = k_{p} \left( \frac{y}{\kappa _{M}+y} - z\right) \\ \end{array} \right. $$

### A short time scale

As with the linear model we use the dimensionless time $\tau _1 = k_{20}t$. Introducing this variable into the system (63) and setting $x=1$, we obtain the reduced system

65 $$ \left\{ \begin{array}{l} \frac{dy}{d\tau _1} = 1- y- \beta \left( \frac{y}{\kappa _{M}+y} - z\right) \\ \frac{dz}{d\tau _1} = \varepsilon \left( \frac{y}{\kappa _{M}+y} - z\right) \\ \end{array} \right.$$

Since $\varepsilon \ll 1$ it follows that for $\tau _1 = O(1)$, we may put $z(\tau _1)=0$ and write the first equation in (65) as an equation for *y* only:

66 $$\begin{aligned} \frac{dy}{d\tau _1} = f(y) \buildrel \rm def \over =1- y- \beta \frac{y}{\kappa _{M}+y} \end{aligned}$$

Since *f*(*y*) is strictly decreasing, $f(0)=1$ and $f(1)<0$, it follows that *f*(*y*) has a unique zero $\overline{y}$ between 0 and 1. Plainly, $\overline{y}$ is one of the two roots of the quadratic equation

67 $$\begin{aligned} y^2 +(\beta +\kappa _M-1)y-\kappa _M=0 \end{aligned}$$

One of these roots is negative and the other is positive. Plainly $\overline{y}$ is the positive one which is given by

68 $$\begin{aligned} \overline{y}=\frac{1}{2}\left\{ (1-\beta -\kappa _M) + \sqrt{(1-\beta -\kappa _M)^2+4 \kappa _M}\right\} \end{aligned}$$

Because $y(0)=0$ and $f(y) >0$ for $0 \le y < \overline{y}$, it follows from (66) that

69 $$\begin{aligned} y(\tau _1) \rightarrow \overline{y}\qquad {\text {as}} \qquad \tau _1 \rightarrow \infty \end{aligned}$$

In order to determine the rate of convergence towards $\overline{y}$ we linearise equation (66) at $\overline{y}$. Writing $y=\overline{y}+\eta$, we can write equation (66) as

70 $$\begin{aligned} \frac{d\eta }{d\tau _1} = f(\overline{y}+\eta ) = f'(\overline{y})\, \eta + \rho (\eta )\qquad {\text {where}} \qquad \eta ^{-1}\,\rho (\eta ) \quad {\text {as}} \quad \eta \rightarrow 0 \end{aligned}$$

in which

71 $$\begin{aligned} f'(\overline{y}) \buildrel \rm def \over = \frac{df}{dy}\Big |_{y=\overline{y}}=-1 - \beta \frac{\kappa _M}{(\kappa _M+\overline{y})^2} \end{aligned}$$

Omitting the small rest term $\rho (\eta )$ we conclude that the terminal slope is approximately given by

72 $$\begin{aligned} 1 + \beta \frac{\kappa _M}{(\kappa _M+\overline{y})^2} \end{aligned}$$

Returning to the original variables, this translates into (cf. equation (25)):

73 $$\begin{aligned} \lambda _z = k_{20} + B_{\rm{max}} \, k_p \frac{K_M}{(K_M+\overline{A}_2)^2} \end{aligned}$$

A graph of the terminal slope in the first phase, as it varies with *q* and $B_{\rm{max}}$, is shown in Fig. 6.

#### Example

When $q=5$ we obtain for the parameter values given by Table 1, that $\overline{y}=0.558$, and hence $\overline{A}_2= 279$ mg, and

74 $$\begin{aligned} f'(\overline{A}_2) = - 0.0223 \qquad \Longrightarrow \quad t_{1/2} = \frac{\ln (2)}{|f'(\overline{A}_2)|} = 31\,\, {\text {h}} \end{aligned}$$

which is consistent with what we observe in the simulation shown in Fig. 3 for $q=5$.

### A large time scale

We now use the dimensionless time $\tau _2 = k_{p}t$, which yields the system

75 $$ \left\{ \begin{array}{l} \varepsilon \frac{dy}{d\tau _2} = 1- y- \beta \left( \frac{y}{\kappa _{M}+y} - z\right) \\ \frac{dz}{d\tau _2} =\frac{y}{\kappa _{M}+y} - z \\ \end{array} \right. $$

On the basis of singular perturbation theory (cf. [7, 8, 10]), we may put

76 $$\begin{aligned} 1- y- \beta \left( \frac{y}{\kappa _{M}+y} - z\right) =0 \end{aligned}$$

which yields an expression for *z* in terms of *y*:

77 $$\begin{aligned} z = \varphi (y) \buildrel \rm def \over = \frac{y}{\kappa _{M}+y} + \frac{1}{\beta }(y-1) \end{aligned}$$

When we substitute this expression into the equation for *z* in Eq. (74) we obtain a single equation for the variable *y* only.

78 $$\begin{aligned} \varphi '(y)\times \frac{dy}{d\tau _2} = \frac{1}{\beta }(1-y) \end{aligned}$$

or, when we divide by $\varphi '(y)$,

79 $$\begin{aligned} \frac{dy}{d\tau _2} =F(y) \buildrel \rm def \over =\frac{1}{\beta }\times \frac{1-y}{\varphi '(y)} \end{aligned}$$

The terminal slope (in terms of $\tau _2$) is now given by

80 $$\begin{aligned} \lambda _z = |F'(1)| = \frac{1}{\beta \, |\varphi '(1)|} = \left( 1+\frac{\beta \kappa _M}{(1+\kappa _M)^2}\right) ^{-1} \end{aligned}$$

In terms of the original time *t* this translates into

81 $$\begin{aligned} \lambda _z(q) = k_p \left( 1+\frac{B_{\rm{max}}\, k_p\, K_M\, k_{20}}{(q+K_M\, k_{20})^2}\right) ^{-1} \end{aligned}$$

We conclude from this expression that the terminal slope increases as the infusion rate increases, and that its limiting values values are given by

82 $$\begin{aligned} \lambda _z(q) \rightarrow k_p \qquad {\text {as}} \qquad q \rightarrow \infty \end{aligned}$$

and

83 $$\begin{aligned} \lambda _z(q) \rightarrow k_p \left( 1+\frac{B_{\rm{max}}}{K_M}\frac{k_p}{k_{20}}\right) ^{-1} \qquad {\text {as}} \qquad q \rightarrow 0 \end{aligned}$$

A graph of the terminal slope in the second phase, as it varies with *q* and $B_{\rm{max}}$, is shown in Fig. 4.

## Appendix 3: mathematical analysis: small capacity and rapid exchange

In Appendix 3 we focus on the *small capacity - fast distribution case* (cf. 47 and 48). Thus,

84 $$k_a \gg k_{p} \gg k_{20}$$

We introduce dimensionless variables using $K_M$ as a reference value for the amounts and $1/k_a$ as a reference time. Thus we put

85 $$\begin{aligned} x = \frac{A_1}{K_M}, \quad y = \frac{A_2}{K_M}, \quad z = \frac{A_3}{K_M}, \quad \tau = k_a\,t. \end{aligned}$$

In terms of these new variables the system (3) becomes for $q=0$,

86 $$ \left\{ \begin{array}{l} \frac{dx}{d\tau } = - x \\ \frac{dy}{d\tau } = x - \mu y - \beta \varepsilon \frac{y}{y+1} + \varepsilon z\quad \beta = \frac{B_{\rm{max}}}{K_M}, \quad \varepsilon = \frac{k_p}{k_a}, \quad \mu = \frac{k_{20}}{k_a}\\ \frac{dz}{d\tau } = \beta \varepsilon \frac{y}{y+1} - \varepsilon z \\ \end{array} \right.  $$

Note that for the parameter values of Table 2, used in Fig. 4, we obtain $\beta =10$, $\varepsilon = 0.2$ and $\mu =0.04$.

In order to obtain a first estimate, we put $\mu =0$ and also $\varepsilon =0$, except when $\varepsilon$ is multiplied by $\beta$ since the product $\beta \,\varepsilon =2$ is clearly not small. Solving the first equation we obtain $x(\tau ) = \delta e^{-\tau }$ with $\delta = D/K_M$. Substituting this expression for $x(\tau )$ into the second equation we obtain a single equation for $y(\tau )$:

87 $$\begin{aligned} \frac{dy}{d\tau } = \delta e^{-\tau } - \beta \varepsilon \frac{y}{y+1} \end{aligned}$$

When $y \ll 1$ Eq. (87) may approximated by the equation

88 $$\begin{aligned} \frac{dy}{d\tau } = \delta e^{-\tau } - \beta \varepsilon y \end{aligned}$$

which can be solved explicitly. Since $y(0)=0$ we obtain for the solution

89 $$\begin{aligned} y(\tau ) = \frac{\delta }{\beta \varepsilon }(e^{-\tau }-e^{-\beta \varepsilon \tau }) \qquad (\beta \varepsilon \not =1) \end{aligned}$$

An elementary computation shows that

90 $$\begin{aligned} y_{\rm{max}} = \delta \times \Bigg \{ \begin{array}{ll} (\beta \varepsilon )^{-\beta \varepsilon /(\beta \varepsilon -1)} &\quad if \,\,\beta \varepsilon \not =1\\ e^{-1} &\quad  if\,\, \beta \varepsilon =1 \\ \end{array} \end{aligned}$$

When $y \gg 1$ we scale Eq. (87) and write $y = \delta z$. The resulting equation may then be approximated by

91 $$\begin{aligned} \frac{dz}{d\tau } = e^{-\tau } - \beta \varepsilon \times \delta ^{-1}. \end{aligned}$$

Its solution which starts at the origin is given by

92 $$\begin{aligned} z(\tau ) = 1 - e^{-\tau } - \frac{\beta \varepsilon }{\delta }\, \tau \end{aligned}$$

Returning to *y*, we then deduce that

93 $$\begin{aligned} y_{\rm{max}} = \delta \, \left( 1-\frac{\beta \varepsilon }{\delta }\right) - \beta \varepsilon \, \ln \left( \frac{\delta }{\beta \varepsilon }\right) = \delta - \beta \varepsilon \, \ln \left( \frac{\delta }{\beta \varepsilon }\right) - \beta \varepsilon \quad {\text {as}}\,\,\,\delta \rightarrow \infty . \end{aligned}$$

In terms of the original variables, the expressions (93) and (93) yield the limits given in Eq. (39).

## Footnotes

1 The big *O*-symbol compares the growth of a function, say *f*(*x*), as $x \rightarrow 0$ or $x \rightarrow \infty$ to that of a simple function, say *g*(*x*). Often $g(x) = x^p$, where *p* may be positive or negative. Specifically: $f(x)=O(g(x))$ as $x \rightarrow 0\, (\infty )$ if there exist a constant *M* such that $|f(x)| \le M|g(x)|$ for *x* small (large)

2 We write $f(x) \sim L \times g(x)$ as $x \rightarrow \infty$ when $\lim _{x \rightarrow \infty }\{f(x)/g(x)\} = L$.
